# Closing gaps in the oxygen supply chain in nations with limited resources

**DOI:** 10.11604/pamj.2024.48.55.43770

**Published:** 2024-06-12

**Authors:** Fabrice Humura, Theogene Uwizeyimana, Josiane Kabayundo, Eric Rucogoza

**Affiliations:** 1 University of Oulu, Research Unit of Population Health, Finland, Pentti Kaiteran Katu, Oulun yliopisto, Finland,; 2 University of Global Health Equity, Butaro District, Northern Province, Butaro, Rwanda,; 3 Public Health Department, University of Nebraska Medical Center, Omaha, NE, United States,; 4 Département de Santé Publique, Université Claude Bernard Lyon 1, 43 Boulevard du 11 November 1918, 69622 Villeurbanne Cedex, France

**Keywords:** Oxygen, supply chain, accessibility, preventable deaths, logistics

## Abstract

Oxygen is an essential medication used across all levels of healthcare for conditions such as surgery, trauma, heart failure, asthma, pneumonia, and maternal and child care. Despite its critical importance and inclusion on the World Health Organization’s list of essential medicines, many low- and middle-income countries (LMICs) face significant challenges in providing adequate oxygen supplies. These challenges are exacerbated by the COVID-19 pandemic, which has drastically increased global oxygen demand. This paper examines the current challenges and advancements in the oxygen supply chain within LMICs, focusing on availability, infrastructure, and usage. It highlights the innovative solutions being implemented to improve oxygen access and offers strategic recommendations for enhancing oxygen delivery and maintenance in resource-limited settings.

## Commentary

Oxygen is a basic medication used to care for patients at all levels of the healthcare framework including in surgery, trauma, heart failure, asthma, pneumonia, and maternal and childcare [1]. Oxygen is currently on the World Health Organization´s (WHO) List of Essential Medicines for acutely ill individuals and for people with chronic diseases resulting in hypoxemia [2]. For example, pneumonia alone accounts for 800,000 deaths per year, and it is assumed that 20-40% of these deaths could be prevented with the accessibility to oxygen therapy [1]. The COVID-19 pandemic has increased global demand for oxygen and made the delivery of oxygen supplies more pressing than ever. The need for oxygen has expanded to 1.1 million cylinders in low to middle-income countries (LMICs) alone [3].

Moreover, many LMICs have long chronic funding shortages for oxygen, with 9 in 10 hospitals from the region unable to provide oxygen therapy, leading to over 800,000 annual preventable deaths [4]. Despite the need and challenges, limited studies are available to discuss the current advances and challenges of oxygen supply in limited resource settings. Therefore, this paper aims to critically examine the current challenges and advances in the oxygen supply chain in low-resource settings and advocate for a strategic approach to close the gaps in the supply chain.

**Current challenges in oxygen supply and access in low- and middle-income countries:** the challenges in ensuring an adequate oxygen supply revolve around issues of availability, infrastructure, and usage.

**Availability:** several studies revealed a shortage of essential equipment in healthcare settings in LMICs. These reviewed surveys showed a shortage of basic equipment, including pulse oximeters and oxygen delivery systems such as cylinders or concentrators. For example, in Uganda, one of East African Countries, it was reported that 15 out of 16 private and public facilities had access to oxygen delivery systems at least half of the time [5]. Yet, six of these hospitals were without access to oxygen for over 25% of the time, and one hospital had no access to oxygen. The availability of pulse oximeters was even more restricted [6]. This implies that even though oxygen may be present in facilities, their capacity to identify patients requiring oxygen and adjust its delivery accurately is constrained. These constraints are not unique in Uganda, they are instead common to other LMICs, particularly in public facilities and those offering lower levels of care. For example, 31% of facilities in sub-Saharan Africa have interrupted oxygen availability and 25% have no oxygen supply [5].

**Infrastructure:** effective distribution networks are essential for timely delivery. This includes well-coordinated logistics and storage facilities. However, some notable hospital departments are lacking such an oxygen infrastructure network. Three studies evaluated oxygen infrastructure globally, with data about LMICs extracted from these studies, revealing comparable results. These studies emphasized surgery and anesthesia capacity in terms of oxygen infrastructure. In a survey across 26 LMICs, it was discovered that 21% of facilities conducting cesareans lacked a dependable oxygen supply. Additionally, 26% of those facilities referring patients out did not have any oxygen supply [7]. Oxygen needs effective logistics for transportation which remains complicated in LMICs. Moreover, oxygen concentrators require consistent electricity to operate, which poses a significant challenge for many facilities, with only 35% to 68% in LMICs having uninterrupted access to electricity [8].

**Usage:** delivering oxygen from a cylinder or concentrator to a patient necessitates fundamental equipment, like tubing, to link the system to a patient delivery apparatus such as a face mask or nasal prongs. A well notable study demonstrated that only 34.3% of facilities had at least one face mask and tube set available in LMICs [8].

**Advances in the oxygen supply chain in low to middle-income countries:** promising advancements are being made in systematically identifying the most effective methods for providing oxygen and in developing innovative systems for oxygen delivery and storage in LMICs. Oxygen concentrators have proven to be significantly more cost-effective than cylinders for delivering oxygen without compromising medical benefits. Such implementation would solve the challenge of oxygen availability at health facilities as replacing cylinders with oxygen concentrators has been proven to be cost-effective and easy to maintain [9]. Papua New Guinea, one of the LMICs, best illustrates effective oxygen program implementation. This included providing pulse oximeters, training staff, installing oxygen concentrators, and designing and assessing the feasibility of a solar-powered oxygen system. Innovations like these are essential for improving oxygen availability and other LMICs could emulate Papua New Guinea's example [9].

Moreover, the installation of user-friendly power supply solutions found to be suitable for LMIC health facilities. Countries such as Gambia addressed poor power availability for oxygen supply by installing Mains-power-storage (Mains-PS) and solar-power storage (Solar-PS) systems. These two systems, which can be assembled without specialist facilities or expertise and use readily available components. Additionally, studies found that the oxygen costs using these two systems were comparable to or lower than the existing cylinder supply systems in standardized facilities modeled for the study's settings [6].

Users in the Gambia expressed high satisfaction with the Mains-PS and Solar-PS systems, indicating a general preference for these new systems over the traditional cylinder-based supply. The primary reasons for this preference included the elimination of concerns about running out of oxygen, avoidance of logistical complications and transaction costs associated with procurement, absence of the need to handle large cylinders, and reduced anxiety about cylinder-related hazards [6]. This approach could be relevant in LMICs with difficulty in energy and infrastructure in general. Many countries have long been struggling to improve equitable access to oxygen therapy, and Several groups, such as PATH, USAID, Bill & Melinda Gates Foundation, and the Clinton Health Access Initiative (CHAI), are addressing some of these issues by helping governments provide medical oxygen access. This entails quick evaluations of oxygen requirements, equipment requirements, and a variety of other variables, such as patient demand and demographics, location and climate, and electrical availability. One illustration is CHAI's unmistakable commitment to propelling National strategic plans for therapeutic oxygen in 17 nations in LMICs [7].

Zambia is one example which is increasing production capacity by expanding the number of production facilities. Zambia has teamed up with the private sector to close oxygen supply gaps by boosting manufacturing capacity, luring capital for more plants, and providing funding to enable hospitals to sign contracts with companies [5]. Rwanda is another example of countries taking actions to close the gap in oxygen supply. In collaboration with institutions such as CHAI and the World Bank, Rwanda has increased the oxygen daily production capacity from 115Nm3 in 2020 to 546Nm3 in 2022, equivalent to more than 600% increase since 2020 [8]. Rwanda achieved such a milestone by deploying 26 newly installed Pressure Swing Absorption (PSA) plants, which generate medical-grade oxygen, across key areas nationwide. These plants are currently supplying oxygen to the primary provincial hospitals, which frequently act as referral centers, as well as neighboring health facilities accessed via a cylinder distribution hub. With capacities ranging from 5 to 40 cubic meters per hour, these PSA plants play a crucial role in enhancing oxygen supply in the country [8]. There is still much to be done, and it would be more significant to maintain the oxygen infrastructure so that those in need can continue to receive it through multi-sector cooperation, especially in low- and middle-income countries.

**Recommendations:** firstly, oxygen needs to be made more accessible and available in healthcare facilities that provide care to adults, especially in public and lower-tier health centers. Engaging stakeholders is essential in this process, and conducting needs assessments will ensure that facilities and communities are included in decision-making. This will require a collaborative effort from national, regional, and local governments, ministries of health, policy experts, healthcare workers, and health facility leaders to identify the appropriate oxygen delivery system for each setting and to ensure sufficient resources are available for maintaining these systems. Secondly, health policymakers need to emphasize infrastructure that are cost-effective and able to endure shortage of power in LMICs like oxygen storage systems or reservoirs. Studies clearly showed that concentrators are more cost-effective than oxygen cylinders and these innovations aiming at reducing the cost of devices could be beneficial.

Lastly, LMIC governments need to cooperate with organizations like CHAI in educating and training healthcare workers to use oxygen effectively and appropriately. The ability to identify patients needing oxygen therapy is hindered by a lack of pulse oximeters and sufficient knowledge. Additionally, appropriately starting, adjusting, and stopping oxygen therapy is constrained by insufficient knowledge and training among healthcare workers at all levels [10]. Pulse oximeters should be available at every healthcare facility.

## Conclusion

In summary, ensuring a reliable oxygen supply in LMICs requires addressing availability, infrastructure, and usage challenges. Promising advancements, such as the use of cost-effective oxygen concentrators and the implementation of oxygen storage systems, have shown potential in improving oxygen access. Collaborative efforts involving national and local governments, health ministries, policy experts, and international organizations are crucial to identifying and maintaining appropriate oxygen delivery systems. Furthermore, training healthcare workers and providing essential equipment like pulse oximeters are vital steps towards improving oxygen therapy. Continued innovation and multi-sector cooperation are essential to sustain and expand these efforts, ultimately enhancing healthcare outcomes in LMICs.
